# The mGluR5 antagonist AFQ056 does not affect methylation and transcription of the mutant *FMR1 *gene *in vitro*

**DOI:** 10.1186/1471-2350-13-13

**Published:** 2012-03-07

**Authors:** Elisabetta Tabolacci, Filomena Pirozzi, Baltazar Gomez-Mancilla, Fabrizio Gasparini, Giovanni Neri

**Affiliations:** 1 Istituto di Genetica Medica, Università Cattolica del S. Cuore, Rome, Italy; 2 Novartis Institutes for BioMedical Research, Basel, Switzerland; 3 Istituto di Genetica Medica, Università Cattolica del Sacro Cuore, Largo F. Vito 1, 00168 Rome, Italy

**Keywords:** Fragile X syndrome, AFQ056, mGluR5 inhibitors, DNA methylation, Epigenetic modification

## Abstract

**Background:**

Fragile X syndrome (FXS), the leading cause of inherited mental retardation, is due to expansion and methylation of a CGG sequence in the *FMR1 *gene, which result in its silencing and consequent absence of FMRP protein. This absence causes loss of repression of metabotropic glutamate receptor 5 (mGluR5)-mediated pathways resulting in the behavioral and cognitive impairments associated with FXS. In a randomized, double-blind trial it was recently demonstrated a beneficial effect of AFQ056, a selective inhibitor of metabotrobic glutamate receptor type 5 (mGluR5), on fully methylated FXS patients respect to partially methylated FXS ones.

**Methods:**

To determine whether AFQ056 may have secondary effects on the methylation and transcription of *FMR1*, here we treated three FXS lymphoblastoid cell lines and one normal control male line. A quantitative RT-PCR was performed to assess transcriptional reactivation of the *FMR1 *gene. To assess the methylation status of the *FMR1 *gene promoter it was carried out a bisulphite sequencing analysis.

**Results:**

Both *FMR1*-mRNA levels and DNA methylation were unmodified with respect to untreated controls.

**Conclusions:**

These results demonstrate that the AFQ056 effect on fully methylated FXS patients is not due to a secondary effect on DNA methylation and consequent transcriptional activation of *FMR1*.

## Background

The fragile X syndrome (FXS, MIM #300624), the most common cause of inherited mental retardation, is due to the amplification (> 200 repeats) of a sequence of CGG triplets in the 5' UTR of the *FMR1 *gene, followed by methylation of cytosines, including those of the promoter upstream [1]. Although the coding region of the gene remains intact, the two changes, one structural and one epigenetic, lead to transcriptional silencing, and consequent absence of the FMRP protein, responsible for the manifestations of the syndrome. FMRP is an RNA-binding protein, which inhibits the translation of messenger RNAs (mRNAs), especially within post-synaptic vesicles of the dendritic spines of hippocampal neurons [2]. It has been demonstrated that the absence of FMRP causes an upregulation of metabotropic glutamate receptors 5 (mGluR5)-mediated signalling pathways, which is the probable cause of the behavioural and cognitive impairments observed in FXS patients [3]. In FXS animal models it was demonstrated that many aspects of the phenotype (behavioural abnormalities, learning deficit, altered dendritic spines, macroorchidism) may be due to excessive mGluR5 signalling. Indeed, crossing *Fmr1 *KO mice with heterozygous *Grm5 *KO mice, expressing reduced amounts of glutamate receptors, rescues many of the FXS phenotypes, except for macroorchidism [4]. Consequently, the use of mGluR5 antagonists may represent an effective treatment for many FXS symptoms. The use of 2-methyl-6-(phenylethynyl)-pyridine (MPEP), a prototypic mGluR5 inhibitor, rescues hyperactivity and audiogenic seizures in *Fmr1 *KO [5]. Recently, in a study performed on *Fmr1 *KO mice, AFQ056, a subtype-selective inhibitor of mGluR5, rescued the inhibition of the startle response after a prepulse, while cultured hippocampal neurons showed shortened dendritic spines [6]. A clinical trial was recently performed to assess the safety and tolerability of AFQ056 in FXS patients, as well as its possible beneficial effect on the behavioural phenotype [7]. This randomized, double-blind, placebo-controlled, cross-over study was performed on 30 FXS male subjects. Seven of these, who were carriers of a fully methylated *FMR1 *mutation, with no detectable production of *FMR1*-mRNA, showed a significant improvement of their behaviour, as measured with the ABC-C scale, after treatment with AFQ056, compared to the placebo-treated controls. No response was detected in FXS subjects who carried a partially methylated *FMR1 *full mutation. This unanticipated finding begs the question whether AFQ056 may have an indirect or secondary effect on methylation of the mutant *FMR1 *gene and, consequently, on its transcription.

To answer this question we studied the effect of AFQ056 on *FMR1 *promoter methylation and mRNA production in three FXS lymphoblastoid cell lines with different degrees of DNA methylation (two fully and one partially methylated) and in one normal control line. No demethylation was induced by the treatment with AFQ056 and the levels of *FMR1*-mRNA remained unmodified. These findings support the conclusion that the AFQ056 effect observed in fully methylated patients is not due to a change in the methylation on the *FMR1 *gene, but may result from the interaction of AFQ056 with other, yet unknown, target proteins.

## Methods

Lymphoblastoid cell lines were established by Epstein-Barr virus (EBV) transformation from peripheral blood lymphocytes of a normal control male (WT) and three FXS males, with CGG expansion of 250 (E3), 450 (S1) and 100-960 (MP, premutation/full mutation mosaic) repeats, respectively. Lymphoblasts were grown in RPMI1640 medium supplemented with 10% fetal bovine serum, 2.5% of L-glutamine and penicillin/streptomycin at 37°C with 5% CO_2_. Treatments were done in T75 flask containing approximately 20 × 10^6 ^cells in a volume of 20 ml. The effect of a single treatment with AFQ056 (Novartis) was assessed at various times and concentrations. The drug was added daily at either 1, 10, 100 or 1,000 μM concentration and cells were harvested to extract RNA and DNA after 3 or 8 days from the beginning of the treatment. Control cultures were sham-treated with the drug diluent. As positive controls for the *FMR1 *reactivation, parallel cultures were also treated with 1 μM 5-aza-2-doxycytydine (5-azadC) (Sigma-Aldrich). Cell viability was assessed after 8 days of treatment using NucleoCounter^® ^(Chemometec, Denmark).

Total RNA from treated and untreated cell lines was extracted with the single-step acid phenol method using Trizol (Invitrogen), reverse-transcribed to cDNA by MoMLV-RT (Invitrogen) and used for quantitative fluorescent RT-PCR, using an ABI 7900 HT (Applied Biosystems) [8].

Bisulphite sequencing of the 52 CpG sites of the *FMR1 *promoter region was performed as previously described [8]. A total of 9 clones were sequenced from WT, E3 and MP cell lines before and after treatment with AFQ056.

## Results

**Figure 1:**
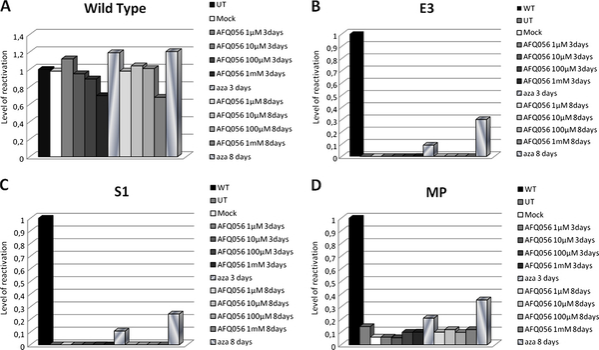
**Quantitative estimate of *FMR1*-mRNA levels in one WT (panel A) and three FXS (panels B-D) cell lines treated for 3 and 8 days with 1, 10, 100 and 1,000 μM AFQ056, respectively**. Real-time fluorescent RT-PCR was normalized to a WT untreated cell line. The same cell lines were also treated with [1 μM] 5-azadC to test the efficiency of transcriptional reactivation and also with the drug diluent (mock). The data are expressed as fractions of the value observed in the untreated WT, arbitrarily set at 1.

The AFQ056 treatment was performed on WT and three FXS lymphoblastoid cell lines. No *FMR1*-mRNA increase was observed after treatment with AFQ056 in any of the four cell lines, with respect to the untreated controls. All results are summarized in Figure 1. The WT cell line was used to establish the basal level of *FMR1 *gene transcription. The partial decrease in *FMR1 *transcription observed in WT at 1 mM AFQ056 after 3 and 8 days of treatment (panel A) was due at least in part to cell mortality. Actually at 8 days post-treatment the cell viability had decreased from 20 to 12 × 10^6 ^cells. A second reason is the physiological fluctuations in *FMR1 *gene transcription previously observed [9]. All cell lines were also treated with [1 μM] 5-azadC for 3 and 8 days, respectively, to test the efficiency of transcriptional reactivation (positive control for *FMR1 *gene reactivation). The 5-azadC showed a mean *FMR1 *reactivation of 10% after 3 days and of 25% after 8 days for E3 and S1 cell lines, while in the WT cell line no significant increase was observed, in accordance with previous results [8]. The MP (size and methylation mosaic) cell line displayed higher levels of *FMR1 *reactivation (20% and 32% after 3 and 8 days, respectively) according with the residual transcription of the gene in these cells. This latter cell line was derived from an FXS boy and did not show any residual translation on Western blot (data not shown).

**Figure 2:**
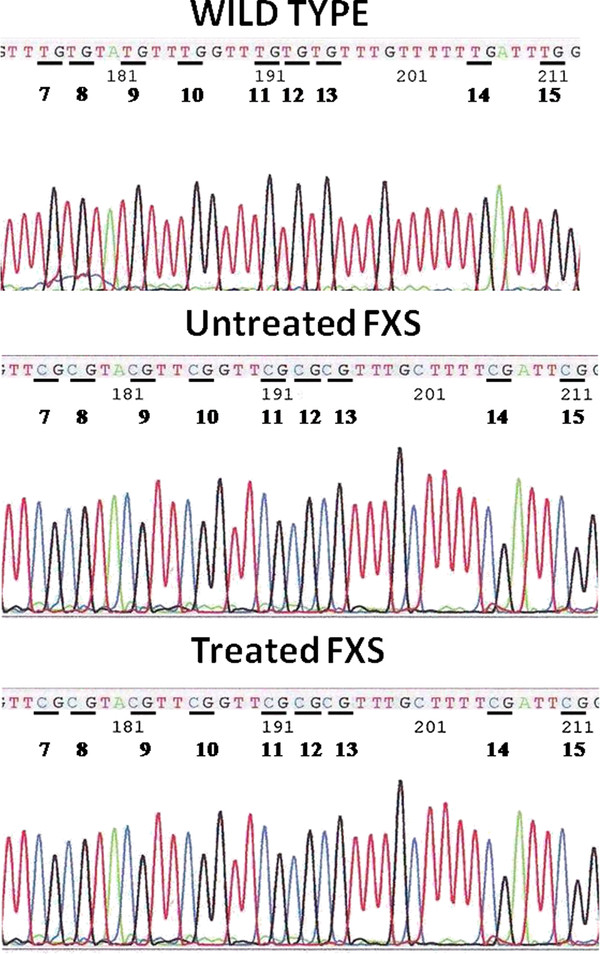
**Partial bisulphite sequence of the *FMR1 *promoter region of a WT (upper panel), untreated FXS (E3 line, middle panel) and treated FXS cell line (E3 + 1 mM AFQ056 for 8 days; lower panel), from CpG site 7 to 15**. After bisulphite treatment, the cytosines of the CpG sites were transformed into thymines in the WT cell line, while in the untreated and treated FXS cells they remained unmodified, demonstrating that AFQ056 treatment does not affect the DNA methylation status in the *FMR1 *promoter region.

In spite of the lack of effect of AFQ056 on *FMR1 *transcript, we went on to analyze DNA methylation of the 52 cytosines constituting the CpG island of the *FMR1 *promoter region. Bisulphite sequencing was performed on 9 clones (cells) of untreated WT and on 4 clones each of WT after 3 and 8 days of treatment with 1 mM AFQ056. A total of 9 clones were sequenced for FXS lines E3 and MP and 9 clones for the same FXS cell lines after treatment. As expected, the promoter of the untreated and treated WT cells was entirely unmethylated, while in the untreated FXS cell lines it was fully methylated, with the exception of few clones completely unmethylated in the MP mosaic cell line. AFQ056 treatment had no effect on methylation, leaving the promoter as methylated as in the untreated controls both WT and FXS (Figure 2).

## Discussion

A link between the neurological and behavioural FXS phenotype, due to absence of FMRP, and the upregulation of mGluR5-mediated activities at dendritic synapses was proposed by the so-called mGluR theory [3]. Both FXS patients and *Fmr1 *KO mice show elongated dendritic spines and enhanced mGluR5-mediated long term depression (LTD) due to a perturbation of the mGluR5 signalling [4].

The observations led to consider the possible use of mGluR5 antagonists to treat FXS symptoms. One of the first compounds to be tested was MPEP, as already discussed [5]. A similar effect was obtained with fenobam, another mGluR5 inhibitor [10]. Fenobam was originally used as an anxiolytic agent, even though at that time its molecular target in the brain was not known. Later on, it was discovered that fenobam is a potent and selective negative allosteric modulator of mGluR5 like MPEP, but with a different chemical structure [11]. A small open-label, single-dose trial of fenobam was conducted to provide an initial evaluation of safety and pharmacokinetics in FXS adults, without any significant adverse effects and with a few beneficial clinical effects [12]. More recently encouraging results were obtained by treating *Fmr1 *KO mice with AFQ056, a subtype-selective inhibitor of mGluR5, capable of rescuing the prepulse inhibition deficit, as well as the dendritic spine phenotype [6]. This compound had previously been used in a clinical trial of Parkinson disease patients with levodopa-induced dyskinesia, demonstrating antidyskinetic effect without changing the antiparkinsonian effects of dopaminergic therapy [13]. Based on evidence of safety and potential clinical efficacy, AFQ056 was used in a randomized, double-blind, two-treatment, two-period, crossover trial of 30 FXS patients aged 18-35 years [7]. In the primary outcome measure (ABC-C score) of this study no statistically significant differences were observed among the treated and untreated groups. However, a secondary exploratory analysis suggested that the response to AFQ056 may be predicted by the methylation status of the *FMR1 *promoter: subjects with a fully methylated *FMR1 *promoter showed statistically significant improvement in their behaviour, while carriers of a partially methylated promoter did not. These findings demonstrated that AFQ056 may alleviate stereotypic behaviour, hyperactivity, inappropriate speech and restricted interests and also improve autistic behaviour in FXS subjects whose *FMR1 *promoter is fully methylated. Our present *in vitro *study of AFQ056 on three FXS lymphoblastoid cell lines with different degree of *FMR1 *promoter methylation now demonstrates that the phenotypic improvement observed in the fully methylated patients is not due to an effect on methylation induced by this drug on the promoter region. Treatment of FXS cell lines with AFQ056 did not cause either an increase in the *FMR1 *transcription or demethylation of the *FMR1 *promoter. These findings were consistently reproducible in three different FXS cell lines. The partial decreased transcription observed in WT cell treated with 1 mM of AFQ056 was probably not due to an effect on DNA methylation, but rather to physiological variations in gene transcription and to cell mortality, as indicated by cell count. Clearly, we are aware that results obtained in lymphoblastoid cells may not necessarily reflect the status of neuronal cells, whose behaviour is central to the pathophysiology of FXS.

## Conclusion

Given the results obtained in this study, one could speculate that full methylation of the *FMR1 *promoter may reflect the activity, or lack thereof, of other proteins interacting with the mGluR5 signalling pathway, thus favouring the beneficial effect of AFQ056 in this subpopulation of FXS patients. An alternative explanation could be that only in the fully methylated patients the mGluR5-mediated signalling is sufficiently high to make the effect of AFQ056 clinically detectable, while this would not be the case in partially methylated patients where the mGluR5 signalling is not so high, due to residual presence of the *FMR1 *protein.

## Competing interests

The authors declare that they have no competing interests.

## Authors' contributions

The original hypothesis regarding the study was conceived by GN. Compound AFQ056 was provided by BGM and FG. Planning of experiments and study design was performed by ET and approved by all authors. Sequencing analysis was performed by FP. The draft was written by GN and ET, all authors revised the manuscript and contributed to the discussion. All authors read and approved the final manuscript.

## Pre-publication history

The pre-publication history for this paper can be accessed here:

http://www.biomedcentral.com/1471-2350/13/13/prepub
